# Cardiac pacing: indications, modalities, complications, and challenges (results of a multicenter cross-sectional study in four hospitals in Dakar, Senegal)

**DOI:** 10.11604/pamj.2024.49.14.43515

**Published:** 2024-09-11

**Authors:** Khadidiatou Dia, Simon Antoine Sarr, Waly Niang Mboup, Youssou Diouf, Nadia Benghazi, Alassane Mbaye, Adama Kane

**Affiliations:** 1 Department of Cardiology, Principal Hospital of Dakar, Dakar, Senegal,; 2 Department of Cardiology, Aristide Le Dantec Hospital, Dakar, Senegal,; 3 Department of Cardiology, Idrissa Pouye Hospital, Dakar, Senegal,; 4 Department of Cardiology, Fann Hospital, Dakar, Senegal

**Keywords:** Cardiac pacing, pacemaker, lead dislodgment, pacemaker infections, reused pacemaker, Senegal

## Abstract

**Introduction:**

cardiac pacing is the only lifesaving procedure which is effective for major cardiac conduction disorders. In sub-Saharan Africa, few pacemakers are implanted, compared to Western countries. This study aimed to describe the indications for cardiac pacing in four hospitals in Senegal, to evaluate its practical modalities, to identify pacemaker’s complications and their predisposing factors and to evaluate the main challenges for cardiac pacing in Senegal.

**Methods:**

we carried out a retrospective study over four years and eight months, from January 2015 to July 2019 in four hospitals in Dakar (Senegal). All patients who received a single-chamber or double-chamber permanent pacemaker were included. Variables included age, gender, symptoms, comorbidities, ECG results, cardiac pacing indications, implantation data, type of procedure, vein approach, use of temporary stimulation lead, data on the characteristics of the pacemaker and probes, and complications.

**Results:**

six-hundred and-twenty (620) permanent cardiac pacemakers were implanted. That is to say an implantation rate of 41 per million population in Senegal. The mean age of the patients was 71±11.77 years. The male gender was in the majority with a sex ratio of 1.19. Eighty-five percent (n=527) of our patients were symptomatic before implantation while 15% (n=93) were asymptomatic. The symptoms were mainly dyspnea in 41% (n=254), dizziness in 32% (n=322) and syncope in 21.3% (n=132). The most found indication was a complete atrioventricular block in 73.7% (n=457), followed by high-degree AVB in 9.2% (n=57). Sinus dysfunction represented 2.4% of indications (n=15). All devices were purchased by patients themselves or their families without government subsidies. Dual-chamber stimulation was the most used mode in 67.1% (n=416) of the patients. Single-chamber stimulation was also used in 32.9% of cases (n=204). The pacemakers were new in 96.1% of cases (n=596) and reused in 3.9% (n=24). The evolution of our patients was generally favorable. Complications occurred in 4.53% of our patients (n=28); mainly leads dislodgements in 1.94% (n=12), infections in 1.29% (n=8), pocket hematomas in 0.65% (n=4), pneumothorax in 0.65% (n=4).

**Conclusion:**

implantations in Senegal are most often salvage implantations with a predominance of complete atrioventricular blocks over sinus dysfunction. Complications of cardiac pacing in our study were mostly lead dislodgment and infections. The challenges facing cardiac stimulation in our country remain the lack of a national registry for implantation database and above all, a lack of accessibility to pacemakers which may be improved by the availability and use of reused pacemakers but also by the introduction of subsidies for cardiac electronic devices by African governments.

## Introduction

Cardiac pacing is the only lifesaving procedure which is effective for major cardiac conduction disorders [1]. In developed countries, nearly 1000 pacemakers are implanted per year per center [2]. In Senegal, compared to Western countries, few pacemakers are implanted [3]. Between 2006 and 2014, nearly 24 pacemakers per year were implanted in one of the main implant centers in Dakar [4]. In 2016, the multicentric study STIMAFRIQUE, carried out across six African countries including Senegal, reported a low implantation prevalence of 2.7 pacemakers/million inhabitants/year [5]. Since then, the activity of cardiac stimulation has been growing in Senegal as reported by only single-center data which reported slightly higher prevalences of cardiac pacing and lesser complications compared with data reported in previous Senegalese studies [6]. It seemed therefore timely for us to conduct a multicentric study with the aim of evaluating cardiac pacing in main reference centers in Senegal, a low-income country. This study aimed to describe the indications for cardiac pacing in four hospitals, to evaluate its practical modalities, to identify pacemaker´s complications and their predisposing factors and to evaluate the main challenges for cardiac pacing in Senegal.

## Methods

**Study design and setting:** the design used in the current study was a cross-sectional hospital-based study design, conducted between January 2015 and July 2019. The study setting was four hospitals in Dakar. The study strictly adhered to the cross-sectional reporting guidelines of Strengthening of Reporting of Observational Studies in Epidemiology (STROBE). This study aimed to describe the indications for cardiac pacing in four public hospitals in Dakar, to evaluate its practical modalities, identify pacemaker complications and their predisposing factors, and evaluate the main challenges for cardiac pacing in Senegal.

**Study population:** the study targeted patients who received a single-chamber or double-chamber permanent pacemaker in the four main hospitals of Senegal whether for a primo-implantation or a change of the pacemaker box. Implantable cardiac defibrillators and triple-chamber pacemakers were excluded from this study. Patients whose files were deemed unusable and those whose follow-up could not be correctly established were excluded. Senegal is located in West Africa, bordering Mauritania to the northeast, Guinea to the southeast, Mali to the East, and the Atlantic Ocean to the West. Senegal is divided into fourteen regions and Dakar is the capital. The main public hospitals of our study are all located in Dakar. These hospitals are the Principal Hospital of Dakar, the Aristide Le Dantec Hospital, the Idrissa Pouye Hospital, and the Fann Hospital. All of these centers have one or two multipurpose angiography rooms where electrophysiological explorations, radiofrequency ablations (in the first three), and interventional cardiology are also carried out. All of these angiographic rooms are dedicated operating blocks with very high-quality image intensifiers, a modern hybrid angiography table, and where the required aseptic conditions are rigorously respected. The number of implanting doctors per center varies between 2 and 5 and in the first three centers there is at least one qualified and graduated rhythmologist.

**Study variables:** variables included age, gender, symptoms, comorbidities (hypertension, diabetes, stroke), ECG results, cardiac pacing indications, implantation data, the type of procedure, the approach, the administered medications, the use of temporary stimulation lead, the existence of intraoperative incidents and accidents), data on the characteristics of the pacemaker and probes, the complications. Variables were summarized and presented in form of frequencies and percentages (Table 1).

**Table 1:** characteristics of the study population, indications and complications

Variable	Total number of cases	%
**Age distribution**		
< 20Y	4	0.6
20-29Y	1	0.2
30-39Y	3	0.5
40-49Y	16	2.6
50-59Y	54	8.7
60-69Y	184	29.7
70-79Y	219	35.3
>80Y	139	22.4
**Symptom**		
Asymptomatic	93	15
Dyspnea/shortness of breath	254	41
Dizziness	201	32.4
Syncope	132	21.3
**Conduction disorders**		
Sinus dysfunction	15	2.4
Third-degree AV block	457	73.7
High-grade AV block	57	9.2
Trifascicular block	2	0.3
Mobitz 2 AV block	12	1.9
Slow rate atrial fibrillation	70	11.3
**Route of pacing**		
Subclavian vein	195	31.5
Cephalic vein	423	68.2
Axillar vein	2	0.3
**Mode of stimulation**		
Single chamber	204	32.9
Dual chamber	416	67.1
**Pacemaker status**		
New	596	96.1
Reused	24	3.9
**Other implantation modalities**		
Primo implantation	562	90.6
Pacemaker box change	58	9.4
Temporary stimulation	139	22.4
**Post-operative complications (4.53%)**		
Lead dislodgment	12	1.94
Infection	8	1.29
Pocket hematoma	4	0.65
Pneumothorax	4	0.65

Y: years; AV: atrioventricular

**Data collection tools:** consisted of a processing sheet. These data came from patient hospitalization records, consultation forms, operating report files, and implantation registers. Demographic variables such as sex and age were collected. Also, the data collection tool, collected variables such as symptoms, comorbidities, pacing indications, implantation data (programmed context or in emergency, primo-implantation or change of pacemaker´s box, the vein approach (cephalic, subclavian or axillar), the use of temporary lead stimulation or not, the characteristics of the pacemaker (new or reused, single or double-chamber, unipolar or bipolar, barbed or active fixation of probes) the occurrence of complications such as lead dislodgement, infection, hematoma, pneumothorax. This monitoring was clinical and based on the search for functional signs, namely syncope and minor equivalents of syncope, dyspnea, and fever. It was also based on local examination of the implantation site next to the box, looking for hematoma, increased local heat, infection, skin erosion, exteriorization of the box, or venous thrombosis. The pacemaker was also controlled using a programmer by affixing the telemetry head to the pacemaker box. Monitoring was also based on paraclinical examinations, by carrying out a standard electrocardiogram (ECG) to check the quality of the detection and stimulation functions of the pacemaker. Chest X-rays were performed to look for pneumothorax, lead displacement, and case migration. Our study focused on the possible occurrence of complications by specifying their type, onset time, treatment methods but also on mortality in our study group. Data were collected and then completed on a survey form.

**Statistical analysis:** to have high-quality data, investigators followed a restricted approach during data collection phase. Variables with missing data were discarded from the analysis. The entry and analysis were carried out respectively on Excel 2013 and SPSS 24.0. For correlations, the Chi-square test and the Fisher test were used, with a significance threshold of p <0.05.

**Ethics approval and consent to participate:** we confirm that for our study, the need for ethics approval was unnecessary, according to national Senegalese regulations. There is presently no legislation in Senegal that requires ethical approval for such studies. Permission was granted from the four hospital directors to carry out this study and access patient information. Data were only accessed by the study team. Confidentiality of the information was assured.

## Results

**General characteristics of the study:** during the period of our study, 890 permanent pacemakers were implanted, but only 620 patients whose follow-up could be well established were included in this study. Two-hundred-and-seven (207) patients were excluded because their implantation and follow-up data were incomplete. The male gender was in the majority with a sex ratio of 1.19. The average age of the patients was 71±11.77 years (from 15 to 98 years). Patients who were over 60 years old represented 85.5% of the population. The majority of patients came from Senegal (94%), mainly from the capital, the 6% came from neighboring African countries (Mauritania, Guinea Conakry, Mali, Gambia, Guinea Bissau, Sierra Leone).

**Clinical features and indications of pacemaker´s implantations:** the majority of our patients, 85% (n=527), were symptomatic before implantation while 15% (n=93) were asymptomatic. Symptoms were mainly dyspnea (41%), dizziness (32.4%), and syncope (21.3%). Indications of implantations were mostly complete atrioventricular block (AVB) for 73.7% of patients (n=457), followed by high-degree AVB in 9.2% (n=57). Sinus dysfunction represented 2.4% of indications (n=15).

**Implantation modalities:** in 90.7% of cases, it was primo implantation, and in 9.3% a pacemaker box change. Temporary stimulation before implantation was used in 22.4% of cases with mean durations of 5 days (1 and 20 days between installation of the temporary stimulator and the definitive stimulation). The cephalic approach was mostly used in 68.3% of cases (n=423), followed by the subclavian approach in 31.5% (n=195) and 0.3% for the axillary route (n=2). Antibiotic prophylaxis based on cefazolin 2 g or, failing that, ceftriaxone 2 g was administered one hour before the incision. Local anesthesia (lidocaine) and analgesic (paracetamol) were administered to all patients at the beginning of the procedure. Pacemakers were new in 96.1% of cases and reused in 3.9%. The cost of one new single-chamber pacemaker was between US$1,480 and US$2,500 and between US$1,800 and US$4,095 for a new double-chamber. These prices excluded operating room and hospitalization costs. All devices were purchased by patients themselves or their families without government subsidies. The right ventricular leads were most often implanted in apical position (98.3%) and in 1.7% in septal position. The right atrial leads were implanted at the side wall of the right atrium (88.2%) and in the auricle 11.8%. After implantations, an oral antibiotic (Cefixime 400 mg per day) was systematically prescribed to all patients.

**Complications of pacemakers and risk factors of complications:** the evolution of our patients was generally favorable. Complications occurred in 28 patients (4.53%), mainly leads dislodgements (1.94%), infections (1.29%), pocket hematomas (0.65%), and pneumothorax (0.65%). Patients who had complications were older than those who had no complications, (72.54±10.91 years vs 71±11.77 years) with a statistically non-significant difference (p=0.49). There was no statistically significant difference between the appearance of complications and the new or reused state of the stimulators (p=0.36). The risk factors which favored the occurrence of complications were: female gender (p=0.042), high blood pressure (p=0.042), use of antiplatelet agents (p=0.0011), and antivitamin K (p=0.006), the existence of intraoperative incidents (p=0.020), the use of the subclavian approach (p=0.05). We did not find a significant correlation between the appearance of complications and the age (p=0.49), the use of a temporary stimulation probe (p=0.55), the diabetic condition (p=0.20), and the presence of underlying heart disease (p=0.23). No correlation was found between probe dislodgement and the use of a barbed probe (p=0.252). Infections occurred in 1.29% of patients with an average onset time of 682 days (22 months 11 days). There was no correlation between the occurrence of infections and the diabetic condition (p=0.632), the existence of intraoperative incidents (p=0.729), the reused state of the pacemaker (p=1), changing the pacemaker box (p=0.102), using a temporary stimulation probe (p=0.11). Pocket hematomas were present in 0.65% of patients. The occurrence of this complication was favored by the presence of arterial hypertension (p=0.042), the use of antiplatelet agents (p=0.0011), the use of vitamin K antagonists (p=0.006), the existence of intraoperative incidents (p=0.04). Pneumothorax occurred in 0.65% of patients who were all implanted through the subclavian vein. Death occurred during follow-up, in 3.87% of patients, mainly in patients who had associated comorbidities (three with stroke, end-stage renal failure, and three with neoplasia).

## Discussion

This study aimed to describe the indications for cardiac pacing in four Senegalese hospitals, evaluate its practical modalities, identify pacemaker complications and their risk factors, and evaluate the main challenges for cardiac pacing in Senegal. Indications were mostly AVB rather than sinus dysfunction. Pacemakers were new in 96.1% of cases and reused in 3.9%. Complications occurred in 28 patients (4.53%), mainly leads dislodgements (1.94%), infections (1.29%), pocket hematomas and pneumothorax (0.65%).

Cardiac pacing is a growing activity in Senegal and other African countries but remains lower than in developed countries [2-9]. This is probably due to the still low number of implanters in our countries and the poor access of populations to pacemakers. Solutions have been found to overcome this problem with the creation in 2017 of the first West-African cardiac Stimulation Inter-University-Diploma initiated by the Gaston-Berger University in Senegal which trains between 5 and 10 African pacemakers implanters every 2 years from various African countries [10]. Besides the human resources which are still lacking, there is the challenge of accessibility to pacemakers by a majority of the population, especially since very few have health coverage or disease insurance. African governments have not yet put in place subsidies on pacemakers which are entirely bought by the patients themselves or the family solidarity helping. Undoubtedly, reused pacemakers may constitute a welcome alternative and solution [5,6,9]. The usual fears regarding these reused pacemakers are infections and dysfunctions of the stimulation system [9], however, in our study and those of many authors, there were no more complications with reused pacemakers [6,9,11]. Access to these reused pacemakers would certainly save lives in our countries knowing that every year, nearly one million people around the world die due to lack of access to pacemakers in low-income countries [12].

Cardiac pacing indications in Senegal are mostly emergency indications of rescue, as complete AVB and high degree AVB are the most common indications compared to sinus dysfunction. It has been also found in African studies [4-6,13-15]. It is known that patients with third-degree AVB who are not implanted have poor survival compared to those who are paced [16,17]. It is different from patients who have sinus dysfunction whose evolution is unpredictable, there is no evidence that the implantation of pacemakers improves the prognosis [18,19] but it makes it possible to significantly improve the quality of life of these implanted patients [20]. However, in developed countries, sinus dysfunction predominates over complete AVB. In a Spanish registry of 12,697 patients, complete AVB represented 23.8% of stimulation indications, less than those for sinus dysfunction at 26.7% [16]. The low proportion of sinus dysfunction in our series (2.4%) was also reported in other African studies between 1.4% and 2.4% [4,6,14]. According to these authors, it may be explained by the lack of awareness of the ECG signs of sinus dysfunction by many physicians and therefore, it may be underdiagnosed [6]. The complications after pacing are the fear of any implanter because they can be serious and difficult to manage [21].

The complications occurred in 4.53% of our study, less than complications reported by the MOST study in the United States of America which were 5.5% at 90 days and 7.5% at 3 years [22]. However, in real life, data seem to indicate a higher risk of possible complications of up to 12.7% in literature data [21,23]. African authors report complications at prevalences ranging from 5.4% to 17.6%) [3,6,11,13,14,24]. The high rate of these complications is predictable given the low annual implantation volume (for the majority less than 50 stimulators implanted per year) compared to Western centers. Data from a large German study show that the annual volume of pacing centers is inversely proportional to the frequency of complications with a large difference between centers that have less than 50 implantations per year and those that have more than 50 [25]. The most frequent complication found in our study is lead dislodgments (1.94%), also found by several African authors as the most frequently encountered complication at proportions that vary between 2.4 and 6.9% depending on the studies [11,14,15].

In another Senegalese series, the most frequently found complication was rather infections 1.3% [6] which are the dread of all stimulators, especially in sub-Saharan Africa where there is still no pacemaker or ICD lead extraction center. This is why in our countries, in the majority of centers, postoperative prophylactic antibiotherapy is systematically instituted with most often Cefixime 400 mg/day. Although this postoperative antibioprophylaxis does not reduce infections according to Western studies [26,27], in our African context with the tropical climatic conditions, the high ambient humidity conducive to the proliferation of germs, the postoperative dressings which are not always carried out in ideal aseptic conditions, all these elements explain that antibiotics are systematically prescribed from the end of the implantation procedure. We found in our work that certain parameters in our study were correlated with the occurrence of complications, notably female gender (p=0.042), and the occurrence of intraoperative incidents (p=0.020). Several studies have found this correlation between the female gender and the appearance of complications. The rate of complications in the women population is significantly higher compared to men. Especially about pneumothorax, pericardial effusions, and pocket hematomas [28]. The possible explanation is that women have a smaller body mass and anatomical differences such as smaller vein sizes and a smaller right ventricle [28].

In the FOLLOWPACE study, men were at less risk of complications compared to women with a hazard ratio of 0.72 [21]. Our study had some limitations inherent to retrospective studies, notably a significant loss of data which led to the exclusion of a huge number of patients, 270 files deemed incomplete. The data was collected from files that were sometimes lost or incomplete with implantation reports sometimes incomplete. All of these lacks show the need and urgency to computerize all data related to procedures and to create national registers in Senegal. This would certainly provide real-life data that better reflects the actual cardiac pacing activity in our countries.

## Conclusion

Cardiac pacing activity remains low in Senegal despite an increase in the number of implantations. Implantations in Africa are most often salvage implantations with a predominance of complete atrioventricular blocks over sinus dysfunction. The challenges facing cardiac stimulation in our regions remain still the lack of national registries for cardiac pacing, insufficient human resources, and above all a lack of accessibility to pacemakers which may be improved by the availability and use of reused pacemakers but also by the introduction of subsidies for cardiac electronic devices by African governments. Despite of all of these challenges which are facing the implanting centers in sub-Saharan Africa, the prevalence of complications remains in most centers within the ranges reported in Western studies, mostly lead dislodgements, infections, pocket hematoma, and pneumothorax.

### What is known about this topic

- Cardiac pacing is a life-saving treatment for major heart conduction troubles;
- In Africa few pacemakers are implanted;
- Pacemakers are expensive in Africa.

### What this study adds

- Implantations in Africa are most often salvage implantations with a predominance of complete atrioventricular blocks over sinus dysfunction;
- Reused pacemakers may be a solution for pacemaker’s poor accessibility;
- Prevalence of complications remains within the ranges reported in Western studies.

## References

[1] The Task Force on cardiac pacing and resynchronization therapy of the European Society of Cardiology (ESC) 2021 (2021). ESC guidelines on cardiac pacing and cardiac resynchronization therapy. Eur Heart J.

[2] Kusumoto FM, Schoenfeld MH, Barrett C, Edgerton JR, Ellenbogen KA, Gold MR (2019). 2018 ACC/AHA/HRS guideline on the evaluation and management of patients with bradycardia and cardiac conduction delay: a report of the American College of Cardiology/American Heart Association Task Force on Clinical Practice Guidelines and the Heart Rhythm Society. J Am Coll Cardiol.

[3] Jouven X, Diop BI, Narayanan K, Adoubi A, Ba SA, Balde D (2019). Cardiac Pacing in Sub-Saharan Africa. J Am Coll Cardiol.

[4] Mbaye A, Fall M, Ngaïdé Aa KI, N´diaye M, Cissé AF (2016). Pratique de la stimulation cardiaque: à propos de 215 implantations de pacemakers au service de cardiologie de l´hôpital général de grand Yoff de Dakar au Sénégal. Cardiologie Tropicale.

[5] Kane A, Houndolo R, Adoubi I, Camara S, Pessinaba S, Sy K (2016). Problematique de la stimulation cardiaque définitive en Afrique sub-saharienne: étude multicentrique STIMAFRIQUE. Cardiol Trop.

[6] Kane A, Sarr SA, Ndobo JVD, Tabane A, Babaka K, Aw F (2019). Cardiac pacing challenge in Sub-Saharan Africa environment: experience of the Cardiology Department of Teaching Hospital Aristide Le Dantec in Dakar. BMC Cardiovasc Disord.

[7] Falase B, Sanusi M, Johnson A, Akinrinlola F, Ajayi R, Oke D (2013). Analysis of a five-year experience of permanent pacemaker implantation at a Nigerian teaching hospital: need for a national database. Pan Afr Med J.

[8] Mond HG, Proclemer A (2011). The 11th world survey of cardiac pacing and implantable cardioverter-defibrillators: calendar year 2009-a World Society of Arrhythmia´s project. Pacing Clin Electrophysiol.

[9] Sani MU, Mayosi BM (2017). The Pacemaker and ICD Reuse Programme of the Pan-African Society of Cardiology. Heart.

[10] Kane A, Adoubi A, Niakara A, Houenassi M, Mbaye A, Sarr SA (2020). Diplôme universitaire de stimulation cardiaque en Afrique sub-saharienne: un bel exemple de coopération Nord-Sud et Sud-Sud. Presse Med Formation.

[11] Adoubi KA, Coulibaly I, Ndjessan JJ, Gnaba A, Tano M, Tro G (2022). Characteristic and evolution of pacemaker complications in a Subsaharan Africa Heart Center. Ann Cardiol and Angeiol. Ann Cardiol Angeiol (Paris).

[12] VanArtsdalen J, Goold SD, Kirkpatrick JN, Goldman E, Eagle K, Crawford T (2012). Pacemaker reuse for patients in resource poor countries: is something always better than nothing. Prog Cardiovasc Dis.

[13] Onakpoya UU, Ojo OO, Eyekpegha OJ, Oguns AE, Akintomide AO (2020). Early experience with pacemaker implantation at a tertiary hospital in Nigeria. Pan Afr Med J.

[14] Camara S, Cheikh M, Ba H, Boye KI, Barry F, Eba A (2019). Profil clinique et suivi des patients porteurs de stimulateurs cardiaques définitifs en Mauritanie. Revue Tunisienne de Cardiologie.

[15] Camara Y, Ba HO, Sangaré I, Thiam CA, Sonfo B, Sidibé N (2021). La Stimulation Cardiaque Définitive au Centre Hospitalier Universitaire Sidi Bocar Sall de Kati: Profil des Patients et Indications. Health Sciences and disease.

[16] Cano Pérez Ó, Pombo Jiménez M, Fidalgo Andrés ML, Lorente Carreño D, Coma Samartín R (2017). Spanish Pacemaker Registry. 14th Official Report of the Spanish Society of Cardiology Working Group on Cardiac Pacing (2016). Rev Esp Cardiol (Engl Ed).

[17] Edhag O, Swahn A (1976). Prognosis of patients with complete heart block or arrhythmic syncope who were not treated with artificial pacemakers. A long-term follow-up study of 101 patients. Acta Med Scand.

[18] Shaw DB, Holman RR, Gowers JI (1980). Survival in sinoatrial disorder (sick-sinus syndrome). Br Med J.

[19] Sutton R, Kenny RA (1986). The natural history of sick sinus syndrome. Pacing Clin Electrophysiol.

[20] Lamas GA, Lee KL, Sweeney MO, Silverman R, Leon A, Yee R (2002). Ventricular pacing or dual-chamber pacing for sinus-node dysfunction. N Engl J Med.

[21] Udo EO, Zuithoff NP, van Hemel NM, de Cock CC, Hendriks T, Doevendans PA (2012). Incidence and predictors of short and long-term complications in pacemaker therapy: THE FOLLOWPACE study. Heart Rhythm.

[22] Ellenbogen KA, Hellkamp AS, Wilkoff BL, Camunãs JL, Love JC, Hadjis TA (2003). Complications arising after implantation of DDD pacemakers: the MOST experience. Am J Cardiol.

[23] Poole JE, Gleva MJ, Mela T, Chung MK, Uslan DZ, Borge R (2010). Complication rates associated with pacemaker or implantable cardioverter-defibrillator generator replacements and upgrade procedures: results from the REPLACE registry. Circulation.

[24] Millogo GR, Seghda A, Ilboudo M, Konaté L, Bassolet B, Kologo JK (2017). Bilan de cinq ans de stimulation cardiaque dans deux structures hospitalières publiques du Burkina Faso: expérience d´une collaboration avec deux centres hospitaliers d´Auvergne. Ann Cardiol Angeiol.

[25] Nowak B, Tasche K, Barnewold L, Heller G, Schmidt B, Bordignon S (2015). Association between hospital procedure volume and early complications after pacemaker implantation: results from a large, unselected, contemporary cohort of the German nationwide obligatory external quality assurance programme. Europace.

[26] Madadi S, Kafi M, Kheirkhah J, Azhari A, Kiarsi M, Mehryar A (2019). Postoperative antibiotic prophylaxis in the prevention of cardiac implantable electronic device infection. Pacing Clin Electrophysiol.

[27] Krahn AD, Longtin Y, Philippon F, Birnie DH, Manlucu J, Angaran P (2018). Prevention of arrhythmia device infection trial: the PADIT trial. J Am Coll Cardiol.

[28] Mohamed MO, Volgman AS, Contractor T, Sharma PS, Kwok CS, Rashid M (2020). Trends of Sex Differences in Outcomes of Cardiac Electronic Device Implantations in the United States. Can J Cardiol.

